# *Ab-Initio* Study of the Electronic and Magnetic Properties of Boron- and Nitrogen-Doped Penta-Graphene

**DOI:** 10.3390/nano10040816

**Published:** 2020-04-24

**Authors:** Chao Zhang, Yu Cao, Xing Dai, Xian-Yong Ding, Leilei Chen, Bing-Sheng Li, Dong-Qi Wang

**Affiliations:** 1 State Key Laboratory of Mining Response and Disaster Prevention and Control in Deep Coal Mines, Anhui University of Science and Technology, Huainan 232001, China; 2 School of Materials Science and Engineering, Anhui University of Science and Technology, Huainan 232001, China; 3 State Key Laboratory for Environment-friendly Energy Materials, Southwest University of Science and Technology, Mianyang 621010, Sichuan, China; 4 Institute of Quantitative Biology and Medicine, SRMP and RAD-X, Soochow University, Suzhou 215123, China; 5 Multidisciplinary Initiative Center, Institute of High Energy Physics, Chinese Academy of Sciences, Beijing 100049, China

**Keywords:** penta-graphene, first-principles calculations, electronic structure, magnetic properties

## Abstract

First-principles calculations were performed to investigate the effects of boron/nitrogen dopant on the geometry, electronic structure and magnetic properties of the penta-graphene system. It was found that the electronic band gap of penta-graphene could be tuned and varied between 1.88 and 2.12 eV depending on the type and location of the substitution. Moreover, the introduction of dopant could cause spin polarization and lead to the emergence of local magnetic moments. The main origin of the magnetic moment was analyzed and discussed by the examination of the spin-polarized charge density. Furthermore, the direction of charge transfer between the dopant and host atoms could be attributed to the competition between the charge polarization and the atomic electronegativity. Two charge-transfer mechanisms worked together to determine which atoms obtained electrons. These results provide the possibility of modifying penta-graphene by doping, making it suitable for future applications in the field of optoelectronic and magnetic devices.

## 1. Introduction

Nowadays, a large variety of experimental and theoretical works have been focused on two-dimensional (2D) materials [1,2,3,4,5,6,7,8,9,10]. One of such examples is graphene [1], which has a two dimensional hexagonal network with unique electrical and mechanical properties [2]. However, because graphene is a semimetal with zero band gap, which greatly limits its applications in electronic devices, a number of additional 2D carbon allotropes have been hotly explored. Zhang et al. recently predicted a new carbon allotrope, penta-graphene (PG) [11], which was composed of only pentagons. Owing to its exceptional properties, such as ultrahigh ideal strength under biaxial strain, negative Poisson’s ratio and the finite electronic band gap, PG is anticipated to be a versatile material for lots of promising applications [12,13,14,15,16,17,18,19].

Moreover, functionalizing 2D materials, for changing their band gaps and manipulating their properties for targeted applications, have received considerable attention. The properties of PG, similar to other 2D materials, can be modulated by structural modifications, mechanical strain, chemical functionalization, and substitutional doping. Substitutional doping is carried out by introducing heteroatoms into the crystal lattice. It has been confirmed that a suitable dopant can alter the physical and chemical properties of carbon nanomaterials and achieve desirable effects, such as high chemical activity, unusual electronic transport properties, and unique optical characteristics [20,21,22]. Boron (B) and nitrogen (N) atoms are typical substitutional dopants in carbon nanomaterials [20,21] owing to their ability to introduce electrons and/or holes into the materials to alter the electronic properties of the materials. Berdiyorov et al. [23] investigated the structure, electronic properties and partial charges of PG substitutionally doped by B, N and Si based on density functional theory (DFT) [24,25]. They found that the electronic bandgap of PG could be reduced to 0.2 eV due to carbon substitutions. Sathishkumar et al. [26] completed a comprehensive study based on DFT computations to provide insight into the dissociation mechanism of hydrogen molecule on pristine, B-, and N-doped PG. They found that the adsorption energy of the H_2_ molecule and H atom on the substrate can be substantially enhanced by incorporating boron or nitrogen into the PG sheet. However, so far, the differences between the effects of only B/N doping at sp^2^ and sp^3^ sites on the electronic structure have not been discussed in detail. This work focuses on the differences in the effects of B/N doping on the electronic structure at these two sites.

In the present study, we explored the effects of substitutional B- and N-doped PG on the electronic and magnetic properties of PG through the ab-initio density functional theory approach. The formation energies of the doping systems were calculated to be between −1.02 and 1.75 eV depending on the type and location of the substitution. Moreover, the electronic band gap of penta-graphene was modulated by changing the type and location of the substitution. The B/N-doped PG system introduced a highly localized intermediate energy level formed in the forbidden band, exhibiting a beneficial *p*-type/*n*-type doping semiconductor characteristic. Furthermore, the introduction of dopant caused spin polarization and led to the emergence of local magnetic moments. The main sources of magnetic moments were analyzed and discussed in detail. These results provide the possibility to modify penta-graphene by doping to make it suitable for future applications in the fields of optoelectronic and magnetic devices.

## 2. Computational Models and Methods

**Figure 1:**
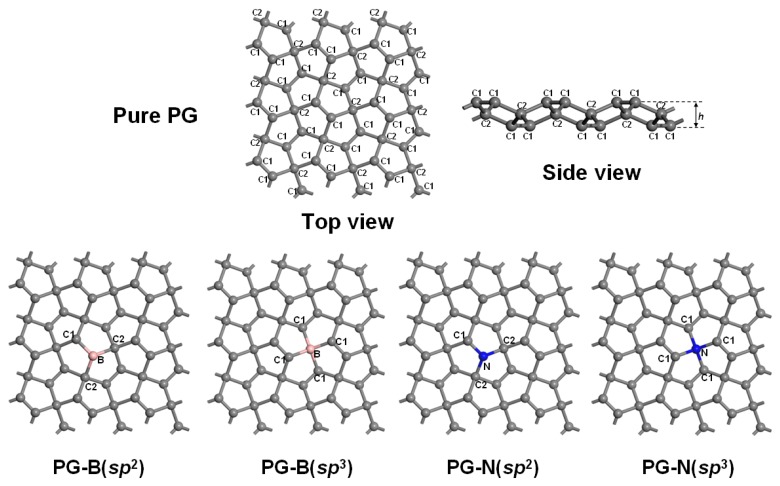
Structures of pristine penta-graphene (PG) and B/N-doped PG model systems. C1 and C2 represent the carbon atoms with sp^2^ and sp^3^ hybridization, respectively, and *h* is the thicknesses of the system. The gray, pink and blue balls represent the carbon, boron, and nitrogen atoms, respectively.

The two-dimensional PG structure possesses *P*-42_1_*m* symmetry and can be described as a tetragonal lattice with six carbon atoms per unit cell. The thickness of this two-dimensional sheet is about 1.2 Å. The carbon atoms in this structure have sp^2^ and sp^3^ hybridizations, and are denoted as C1 and C2, respectively. In the present work, the pure PG supercell with 54 atoms consists of 3 × 3 × 1 primitive cells. Four types of B- and N-doped PG systems are constructed by substituting a C1/C2 atom with a B/N atom, marked by PG–B (sp^2^), PG–B (sp^3^), PG–N (sp^2^) and PG–N (sp^3^), respectively, as shown in Figure 1.

The ab-initio density-functional theory (DFT) calculations are performed using the Cambridge Sequential Total Energy Package (CASTEP) code [27,28,29,30]. The generalized gradient approximation (GGA) of Perdew–Burke–Ernzerhof (PBE) functional is employed to describe the exchange and correlation interactions [31,32]. The electron-core interaction is described by using the ultrasoft pseudopotential [33]. The periodic boundary condition is imposed in the *x*- and *y*-dimensions, and a vacuum layer with a thickness of 15 Å is applied in the *z*-dimension to prevent virtual interactions. For geometry optimization, an energy cutoff of 500 eV for the plane-wave basis set is applied to expand the electronic wave functions. The Brillouin zone is sampled by 11 × 11 × 1 Monkhorst-Pack special *k*-point mesh for structural optimization and electronic structural calculations. The convergence criteria for the total energy and the Hellman–Feynman forces are set to be 10^−5^ eV/atom and 10^−2^ eV/Å, respectively.

To examine the structural stability and doping efficiency of B/N-doped PG systems, we calculate the formation energy (*E*_f_) for the B/N-doped PG systems, defined by the following equation:

*E*_f_ = *E*_dop_ − *E*_pris_ − *E*_B/N_ + *E*_C_ (1)

where *E*_dop_ and *E*_pris_ are the total energies of the B/N-doped PG and pristine PG systems with the same size supercell, respectively. *E*_B/N_ and *E*_C_ are the chemical potential of B/N and C atoms, respectively. *E*_C_ is obtained via dividing the energy of the pristine PG by 54. *E*_B_ and *E*_N_ are obtained through calculating the energy of the elementary substance with the best thermodynamic stability, i.e., α-boron and N_2_, respectively, and dividing the number of atoms in elementary substance.

## 3. Results

**Figure 2:**
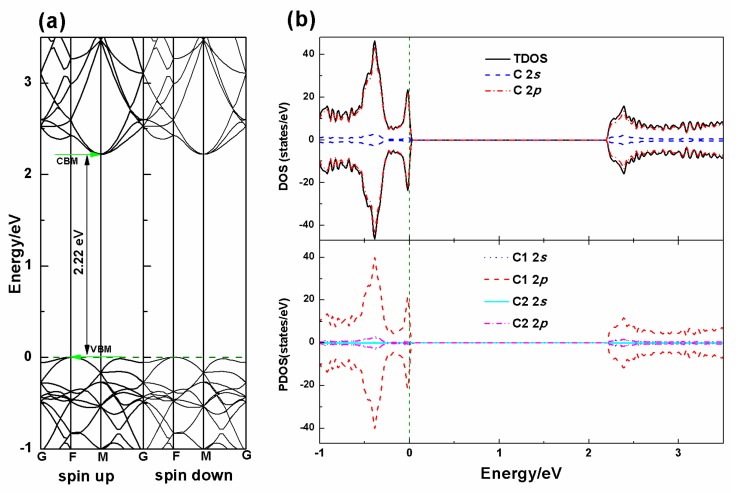
Calculated electronic energy band structure (**a**), total density of states (TDOS) and partial density of states (PDOS) of the pure PG (**b**). On the top layer of (**b**), the black solid line in the density of states (DOS) plot shows the TDOS of the system, and the blue dash and the red dash dot lines indicate the PDOS of the 2s and 2p states of carbon atoms, respectively. On the bottom layer of (**b**), the blue dot and the red dash lines indicate the PDOS of 2s and 2p states of C1 atoms, respectively, and the cyan solid and the magenta dash dot lines indicate the 2s and 2p states of C2 atoms, respectively. The olive dash lines represent the Fermi energy level. Valence band maximum (VBM) and conduction band minimum (CBM) indicate the valence band maximum and the conductance band minimum, respectively.

In order to benchmark the theoretical method employed and test the validity of the parameters, the pristine PG supercell is first optimized and the obtained results are shown in Table 1. The calculated lattice parameters are *a* = *b* = 10.904 Å with a thickness of 1.203 Å. The C1–C1 and C1–C2 bond lengths are 1.337 and 1.547 Å, respectively, which are consistent with the previous published results [11,34]. The electronic band structure of the optimized PG supercell is also calculated and shown in Figure 2. A quasi-direct band gap of 2.22 eV is observed, which agrees with the previous studies [34]. The analyses of the total density of states (TDOS) and partial density of states (PDOS) of the PG, as shown in Figure 2b, indicate that the TDOS is mainly attributed to the C1 2p states, with little C2 2p states. As can be seen from Figure 1, C1 and C2 atoms have a different chemical environment with saturating 3 and 4 bonds, respectively, corresponding to sp^2^ and sp^3^ hybridization modes, which determines their contribution to the DOS. The atomic Hirshfeld charge, which is a relatively accurate way of describing atomic charge, and the magnetic moment for the pristine PG system are also calculated, and it is found that there is no magnetic moment in the pure PG system with the C1 atoms negatively charged (−0.01|e|) and the C2 atoms positively charged (0.02|e|), indicating an electronic structure with discretized, rather than uniformed, charge distribution.

### 3.1. Geometrical Structure and Stability

The doped PG systems are optimized, and the obtained results show the lattice parameters for both the PG–B (sp^2^) and PG–B (sp^3^) systems increase: *a* = *b* = 10.920 Å and 10.948 Å, respectively. Such an increase in the lattice parameter is ascribed to the fact that the covalent radius of a boron atom is greater than that of a carbon atom, forming longer B–C bonds. As shown in Table 1, the lengths of B–C1 and B–C2 bonds in the PG–B (sp^2^) system are 1.502 Å (>1.337 Å) and 1.613 Å (>1.547 Å), respectively. It changes to 1.594 Å (>1.337 Å) for the B–C1 bond in the PG–B (sp^3^) system. The larger bond length causes the monolayer to deform and the thickness to become thicker, which is calculated to be 1.394 Å and 1.304 Å, respectively, for the PG–B (sp^2^) and PG–B (sp^3^) systems.

The lattice constants of the PG–N (sp^2^) and PG–N (sp^3^) systems are calculated to be *a* = *b* = 10.873 Å and *a* = 10.873, *b* = 10.871 Å, respectively, indicating that N doping shrinks the PG system. This is because a nitrogen atom has a smaller covalent radius than a carbon atom, resulting in shorter nitrogen–carbon bonds. The bond lengths between N and C2 atoms in the PG–N (sp^2^) system are calculated to be about 1.542 Å, and the N–C1 bond lengths in the PG–N (sp^3^) system are about 1.546 Å, which are smaller than the corresponding values of the pristine PG system (see Table 1). However, the N–C1 bond length in the PG–N (sp^2^) system is about 1.384 Å, which is larger than that of the PG. The reason is that the two shortened N–C2 bonds make the N–C1 bond longer. Although the N–C1 bond is longer, the lattice constant still decreases.

As can be seen from Table 1, the formation energies of PG–B (sp^2^), PG–B (sp^3^), PG–N (sp^2^) and PG–N (sp^3^) are calculated to be about 1.39, 1.75, −1.02 and 1.14 eV, respectively. It is well known that a more negative formation energy corresponds to a more thermodynamically stable geometrical structure. Therefore, the thermodynamic stabilities of the four systems are PG–N (sp^2^) > PG–N (sp^3^) > PG–B (sp^2^) > PG–B (sp^3^). The formation energies in the latter three cases are positive and sizeable, and their preparation may thus require special treatment, e.g., laser ablation.

### 3.2. Electronic Properties

**Figure 3:**
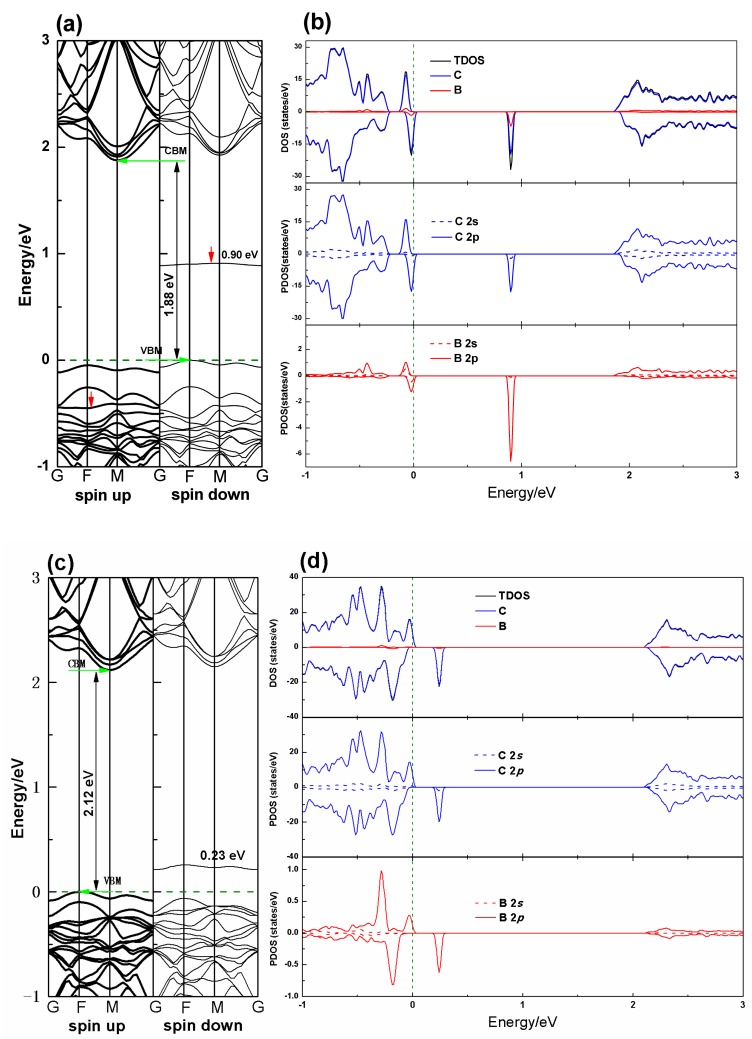
Calculated electronic energy band structure, TDOS and PDOS of the PG–B (sp^2^) ((**a**) and (**b**)) and PG–B (sp^3^) ((**c**) and (**d**)) systems. The black solid line in the density of states plot shows the TDOS of the system. The blue and red lines indicate the PDOS of carbon and boron atoms, respectively. The olive dash lines represent the Fermi energy level.

Figure 3 shows the band structures and densities of states (DOS) of the PG–B (sp^2^) and PG–B (sp^3^) systems. As can be seen from the figure, the band gaps are 1.88 and 2.12 eV, respectively, for PG–B (sp^2^) and PG–B (sp^3^) systems, which are both smaller than that of the PG system. In PG–B (sp^2^) and PG–B (sp^3^) systems, both the valence band (VB) and the conductance band (CB) shift toward the lower energy range, indicating that the substituent B dopant has a significant effect on the band structure of pristine PG. Interestingly, in the electronic structures of the PG–B (sp^2^) system (see Figure 3a), there are two highly localized energy levels near the Fermi level in the spin-up and spin-down states, respectively. It can be concluded from the density of state in Figure 3b that the two energy levels are mainly contributed by the C 2p states, with little contribution from the B 2p states.

There exists a new intermediate energy level formed in the spin-down states in the forbidden band at about 0.90 and 0.23 eV for the PG–B (sp^2^) and PG–B (sp^3^) systems, respectively. Analysis of DOS shows that the intermediate energy level in the PG–B (sp^2^) system is mainly attributed to the hybridization of the spin-down states of carbon and boron atoms (see Figure 3b), i.e., the hybridization of C 2p and B 2p states according to the examination of PDOS. In the PG–B (sp^3^) system, the intermediate energy level is mainly attributed to the spin-down states of C 2p states, with little B 2p states (see Figure 3d). Moreover, there is an intriguing phenomenon discovered from the band structure. In the PG–B (sp^2^) system, the intermediate energy level may be derived from the upshift of the original third energy band below the Fermi level in the spin-down states, compared with the third energy band (marked by red arrow) below the Fermi level in the spin-up states (see Figure 3a), while in the PG–B (sp^3^) system, the intermediate energy level is derived from the upshift of the original first the energy band below the Fermi level in the spin-down states (see Figure 3c). The intermediate energy level divides the wider energy gap into two different narrower band gaps and provides an additional state for the excitation of electrons at the valence band maximum (VBM). The excited electrons could be again excited to the conductance band from the intermediate state. These results indicate both the PG–B (sp^2^) and PG–B (sp^3^) systems are *p*-type behavior due to the electron-deficient nature of B atoms and provide the possibility for tuning the PG band gap by boron doping to make the material suitable for optoelectronic and photovoltaic applications.

**Figure 4:**
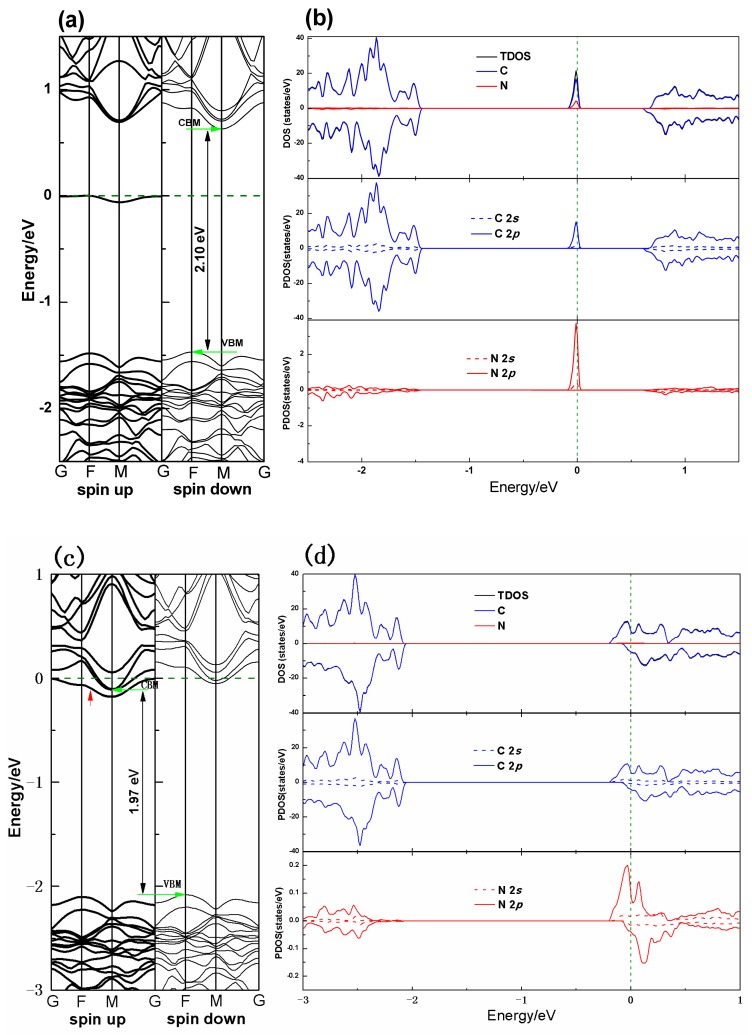
Calculated electronic energy band structure, TDOS and PDOS of the PG–N (sp^2^) ((**a**) and (**b**)) and PG–N (sp^3^) ((**c**) and (**d**)) systems. The black solid line in the density of states shows the TDOS of the system. The blue and red lines indicate the PDOS of carbon and nitrogen atoms, respectively. The olive dash lines represent the Fermi energy level. The energy level marked by the red arrow in the spin-up states in (**c**) is an impurity energy level due to the introduction of the nitrogen atoms.

Figure 4 shows the electronic band structure and the density of states of the PG–N (sp^2^) and PG–N (sp^3^) system, respectively. From the figures, it is found that the Fermi level is shifted upward and toward the conduction band in both the spin-up and spin-down states, and even passes through the conduction band in the PG–N (sp^3^) system (see Figure 4c), which is ascribed to the electron-rich characteristics of nitrogen atoms. The band gap is 2.10 and 1.97 eV, respectively for the PG–N (sp^2^) and PG–N (sp^3^) systems, which are smaller than that of PG system. There is a local impurity energy level appeared near the Fermi surface in the spin-up states in the PG–N (sp^2^) system (see Figure 4a), which is ascribed to the hybridization of the spin-up states of carbon and nitrogen atoms according to the DOS plot (see Figure 4b). Further examination of the PDOS shows that the doped energy level is mainly derived from the hybridization of C 2p and N 2p states. The doping energy level divides the wider energy gap into two different narrower band gaps and therefore enhance the conductance of PG. In the PG–N (sp^3^) system, the doping energy level is marked by the red arrow in the spin-up states (see Figure 4c), which is so close to the conduction band that the Fermi level passes through the conduction band. The result indicates that the PG–N (sp^2^) system is *n*-type behavior of N doping due to the electron-rich characteristics of N atoms.

**Figure 5:**
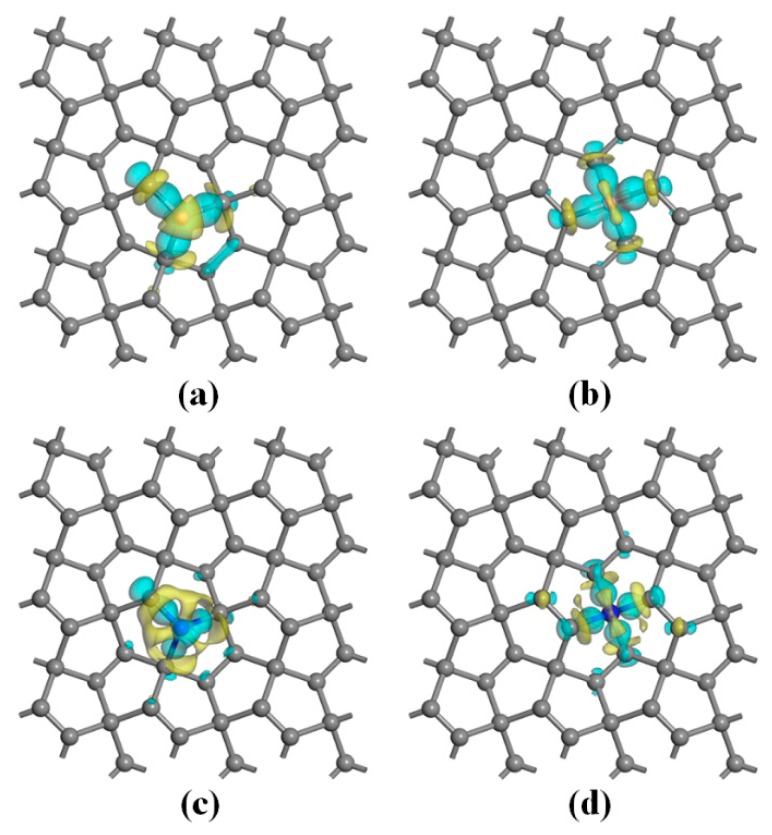
Isosurface plots of electron density differences for the (**a**) PG–B (sp^2^), (**b**) PG–B (sp^3^), (**c**) PG–N (sp^2^) and (**d**) PG–N (sp^3^) systems with the isovalues of ± 0.06 a.u. The cyan and yellow represent charge accumulation and depletion, respectively.

The isosurface plots of electron density differences of the four system are displayed in Figure 5. It can be seen from the figure that the charge accumulation indicates the chemical bonds between dopants and carbon atoms have strong covalent bond characteristics. For B doping, no matter the PG–B (sp^2^) or PG–N (sp^3^) system, there is more charge depletion in the vicinity of B atoms relative to carbon atoms, indicating that boron atoms contribute more electrons relative to carbon atoms, that is, B atoms act as donors, which are consistent with the impurity levels below the conduction bands but above the Fermi level presented in Figure 3a,c. For N doping, the connected carbon atoms have more obvious charge depletion compared with N atoms, indicating that N atoms act as acceptors, which are consistent with the result that the doping level is below the Fermi level in the energy band structure (see Figure 4a,c). In addition, it can be found that the charge accumulation in the N–C bond regions of the PG–N (sp^2^) system is stronger than that of the PG–N (sp^3^) system, and the charge depletion is observed in the off-bonding region of PG–N (sp^2^) system (Figure 5). These results indicate the attenuation of electronic delocalization, i.e., the enhancement of electronic localization.

### 3.3. Magnetic Properties

**Figure 6:**
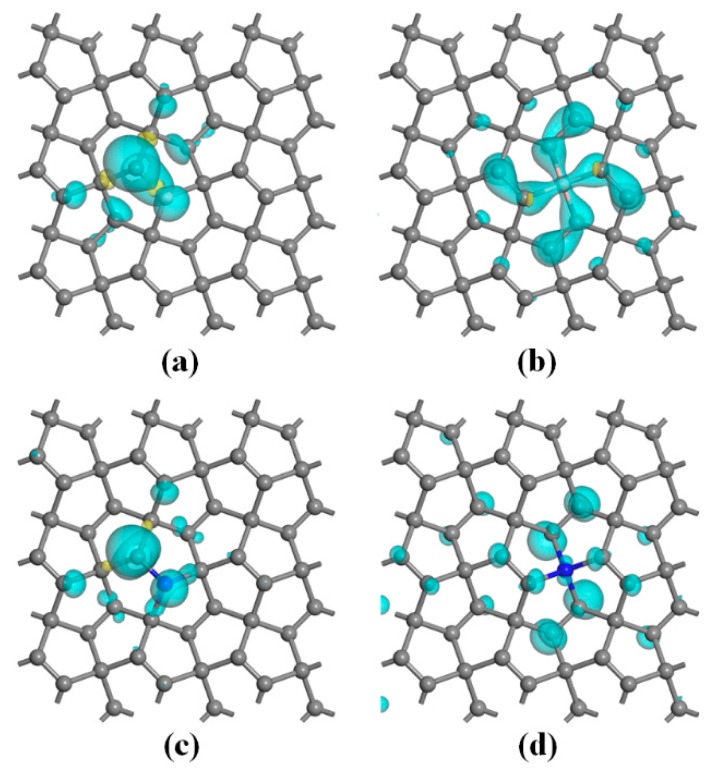
Spin distributions of (**a**) PG–B (sp^2^), (**b**) PG–B (sp^3^), (**c**) PG–N (sp^2^) and (**d**) PG–N (sp^3^) with the isovalues of ± 0.02 a.u. The poor blue and yellow represent positive and negative spin densities, respectively.

The spin distributions are presented to explore the distribution of the magnetism. The spin-polarized charge density of the PG–B (sp^2^) and PG–B (sp^3^) systems are analyzed and the isosurfaces are depicted in Figure 6a,b, respectively. The magnetization for the PG–B (sp^2^) system is observed to distribute symmetrically along the B–C1 bond and is mainly located on the boron atom and the nearest neighboring C1 atom, with little on other adjacent carbon atoms (see Figure 6a). For the PG–B (sp^3^) system, the magnetization is centrosymmetric distribution centered on the B atom and is mainly locates on the B dopant and its nearest and second neighboring C1 atoms (see Figure 6b). The main reasons for these phenomena are as follows. In the PG–B (sp^2^) system, after a B atom is doped at a C1 (sp^2^–C) site, the original *π* bond formed between two C1 atoms is opened to form an unpaired electron on another C1 atom and an empty *pz* orbital on replacement cite due to the sp^2^ hybridization for the B atom, causing the unpaired electron to polarize to the empty *pz* orbital, as shown in Figure 6a. This leads to a net magnetic moment of 0.16 μ_B_ on the B atom and 0.56 μ_B_ on the C1 atom, respectively, as shown in Figure S1. Therefore, the total magnetic moment of the system is mainly contributed by the C1 and B atoms. Further research shows that the direction of charge transfer between the boron and carbon atoms may be attributed to the combined effects of the following two aspects. On the one hand, because the carbon atom is more electronegative than the boron atom, the B atom transfers little charge to the adjacent carbon atoms during the formation processes of the *σ* bonds. On the other hand, the unpaired electron on the C1 atom polarizes little charge to the *pz* orbital. Two charge-transfer mechanisms work together to determine which atoms obtain electrons. Figure S2a shows the atomic charge of the PG–B (sp^2^) system. It is found from the figure that the B atom is positively charged (+0.15|e|) and the adjacent C1 atom is negatively charged (−0.03|e|), indicating the B atom transfers little charge to the adjacent C1 atom. In this case, the B–C1 bond (1.502 Å) is a σ single bond with a little *π* bond trend, so it should be a little shorter than a B–C2 single bond (1.613 Å), as shown in Table 1.

In the PG–B (sp^3^) system, the boron atom is sp^3^ hybridized with four C1 atoms. Since a boron atom has only three valence electrons, it needs to obtain electrons from the surrounding carbon atoms to form four single bonds. As shown in Figure 6b, the four nearby C–C *π* bonds transfer electrons to the B atom. As a result, there are fewer electrons in the *π* bonds, leading to a net spin. The total spin of the system is mainly contributed by the four *π* bonds. Since the spin is attached to the atom, there is a significant spin on the nearby eight carbon atoms. Hence, the total magnetic moment of the system is mainly contributed by the B dopant (0.04 μ_B_) and its nearest (0.06 μ_B_) and second neighboring (0.1 μ_B_) C1 atoms. Similar to the PG–B (sp^2^) system, the boron atom also transfers electrons to surrounding carbon atoms due to the different electronegativity. The atomic hirshfeld charge on the boron atom is calculated to be zero, as shown in Figure S2b, which indicates that the two mechanisms transfer the same amount of charge. Since the nearby *π* bonds transfer electrons to the boron atom to form B–C bonds, the bond lengths (1.348 Å) of the nearby C=C double bonds are larger than those (1.337 Å) of pristine PG (See Figure S3).

Besides, it can be inferred from the DOS that N dopant makes the PG system create spin polarization phenomenon. Detailed analysis of spin density, which is defined as the difference between the Alpha and Beta electron density, i.e., Δ*ρ* = *ρ* (alpha) − *ρ* (beta), shows that the magnetic moment in the PG–N (sp^2^) system is mainly located on the nitrogen atom and the nearest neighboring C1 atoms and is symmetry distribution along the N–C1 bond (see Figure 6c). In the PG–N (sp^3^) system (see Figure 6d), the magnetic moment is almost centrosymmetric distribution centered on the N atom and is mainly located on partial C1 atoms, which is dissimilar to that of the PG–B (sp^3^) system. The main reasons for the above phenomena are as follows. In the PG–N (sp^2^) system, the N atom is sp^2^ hybridized with three carbon atoms, forming three single bonds, and there is a lone pair of electrons on the N atom, which is a perfect and stable electronic structure. Therefore, the unpaired electron on the adjacent C1 atom cannot be transferred to the N atom. It can only be transferred to other neighboring carbon atoms and thus the total magnetic moment of this system should be mainly contributed by the C1 atom, and the N atom should have almost no magnetic moment due to its saturated electronic structure. As shown in Figure 6c and Figure S1, the spin magnetic moment of the C1 atom is 0.58 μ_B_, while that of the N atom is only 0.10 μ_B_. The tiny magnetic moment on the N atom may be ascribed to the charge transferred from the carbon atoms due to the difference in electronegativity. Therefore, the N atom is negatively charged (see Figure S2c).

In the PG–N (sp^3^) system, the N atom is sp^3^ hybridized with four carbon atoms. There is an unpaired electron left on the nitrogen atom, which is not stable and thus has to be transferred to the nearby carbon atoms. Therefore, the total magnetic moment of the system is mainly contributed by the C1 atoms and the N dopant atom (see Figure S1). In addition, the N atom should also obtain electrons from the nearby carbon atoms due to the different electronegativity. Two charge-transfer mechanisms work together to determine that there is a positive charge on the nitrogen atom, as shown in Figure S2d.

## 4. Conclusions

In summary, first-principles calculations have been performed to investigate the electronic and magnetic properties of the boron/nitrogen-doped PG systems. The formation energies are calculated to be 1.39, 1.75, −1.02 and 1.14 eV, respectively, which indicates that the thermodynamic stability of the four systems is PG–N (sp^2^) > PG–N (sp^3^) > PG–B (sp^2^) > PG–B (sp^3^). The band gaps of the systems are calculated to be 1.88, 2.12, 2.10 and 1.97 eV, respectively, all of which are in the visible light range and enable their potential applications in photocatalysis.

By analyzing the spin-polarized charge density, we find that, in the PG–B (sp^2^) system, the unpaired electron on the carbon atom attached to the doped B atom polarizes to the empty *pz* orbital, leading to a net magnetic moment of 0.16 μ_B_ on the B atom and 0.56 μ_B_ on the carbon atom, respectively. In the PG–B (sp^3^) system, the total magnetic moment of the system is mainly contributed by the doped B atom (0.04 μ_B_) and its nearest neighbor (0.06 μ_B_) and the next nearest neighbor (0.1 μ_B_) C1 atoms because the four nearby C–C *π* bonds transfer electrons to the B atom. The magnetic moment in the PG–N (sp^2^) system is mainly contributed by the carbon atom attached to the N atom due to the unpaired electron on the carbon atom transferred to the adjacent carbon atoms. The total magnetic moment in the PG–N (sp^3^) system is mainly contributed by the doped N atom and its surrounding carbon atoms due to an unpaired electron left on the nitrogen atom, which is not stable and thus has to be transferred to the nearby carbon atoms. These results indicate that PG material is expected to be applied in the field of magnetic devices in the future through doping modification.

## Figures and Tables

**Table 1:** Lattice parameters (*a* and *b* in Å), thickness (*h*, in Å), lengths of key bonds (*d*, in Å), band gap (*E*_g_, in eV), formation energy (*E*_f_, in eV) of the pure PG, PG–B (sp^2^), PG–B (sp^3^), PG–N (sp^2^) and PG–N (sp^3^) systems.

System	*a* (Å)	*b* (Å)	*h* (Å)	*d* (Å)	*E*_g_ (eV)	*E*_f_ (eV)
**Pure PG**	10.904	10.904	1.203	C1–C1 = 1.337 C1–C2 = 1.547	2.22	-
**PG–B (sp^2^)**	10.920	10.920	1.394	B–C1 = 1.502 B–C2 = 1.613	1.88	1.39
**PG–B (sp^3^)**	10.948	10.948	1.304	B–C1 = 1.594	2.12	1.75
**PG–N (sp^2^)**	10.873	10.873	1.286	N–C1 = 1.384 N–C2 = 1.542	2.10	−1.02
**PG–N (sp^3^)**	10.873	10.871	1.310	N–C1 = 1.546	1.97	1.14

## Supplementary Materials

The following are available online at https://www.mdpi.com/2079-4991/10/4/816/s1, Figure S1: Atomic net magnetic moments (μ_B_); Figure S2: Atomic hirshfeld charge (|e|); Figure S3: Bond lengths (Å).

## References

[1] Novoselov K.S., Geim A.K., Morozov S.V., Jiang D., Zhang Y., Dubonos S.V., Grigorieva I.V., Firsov A.A. (2004). Electronic field effect in atomically thin carbon films. Science.

[2] Pykal M., Jurečka P., Karlický F., Otyepka M. (2016). Modelling of graphene functionalization. Phys. Chem. Chem. Phys..

[3] Ouyang W.G., Mandelli D., Urbakh M., Hod O. (2018). Nanoserpents: Graphene nanoribbon motion on two-dimensional hexagonal materials. Nano Lett..

[4] Singh D., Gupta S.K., Sonvane Y., Hussain T., Ahuja R. (2018). Achieving ultrahigh carrier mobilities and opening the band gap in two-dimensional Si_2_BN. Phys. Chem. Chem. Phys..

[5] An Y.P., Jiao J.T., Hou Y.S., Wang H., Wu D.P., Wang T.X., Fu Z.M., Xu G.L., Wu R.Q. (2018). How does the electric current propagate through fully-hydrogenated borophene. Phys. Chem. Chem. Phys..

[6] Liu C.W., Dai Z.X., Zhang J., Jin Y.G., Li D.S., Sun C.H. (2018). Two-dimensional boron sheets as metal-free catalysts for hydrogen evolution reaction. J. Phys. Chem. C.

[7] Mannix A.J., Zhou X.F., Kiraly B., Wood J.D., Alducin D., Myers B.D., Liu X.L., Fisher B.L., Santiago U., Guest J.R. (2015). Synthesis of borophenes: Anisotropic, two-dimensional boron polymorphs. Science.

[8] Tang X., Li S.S., Ma Y.D., Du A.J., Liao T., Gu Y.T., Kou L.Z. (2018). Distorted Janus transition metal dichalcogenides: Stable two-dimensional materials with sizable band gap and ultrahigh carrier mobility. J. Phys. Chem. C.

[9] Wang X.Q., Li H.D., Wang J.T. (2013). Prediction of a new two-dimensional metallic carbon allotrope. Phys. Chem. Chem. Phys..

[10] Lu H., Li S.D. (2013). Two-dimensional carbon allotropes from graphene to graphyne. J. Mater. Chem. C.

[11] Zhang S.H., Zhou J., Wang Q., Chen X.S., Kawazoe Y., Jena P. (2015). Penta-graphene: A new carbon allotrope. Proc. Natl. Acad. Sci. USA.

[12] Li X., Zhang S., Wang F.Q., Guo Y., Liu J., Wang Q. (2016). Tuning the electronic and mechanical properties of penta-graphene via hydrogenation and fluorination. Phys. Chem. Chem. Phys..

[13] Wu X., Varshney V., Lee J., Zhang T., Wohlwend J.L., Roy A.K., Luo T. (2016). Hydrogenation of penta-graphene leads to unexpected large improvement in thermal conductivity. Nano Lett..

[14] Krishnan R., Wu S.Y., Chen H.T. (2018). Nitrogen-doped penta-graphene as a superior catalytic activity for CO oxidation. Carbon.

[15] Krishnan R., Su W.S., Chen H.T. (2017). A new carbo n allotrope: Penta-graphene as a metal-free catalyst for CO oxidation. Carbon.

[16] Chen M.W., Zhan H.F., Zhu Y.B., Wu H.G., Gu Y. (2017). Mechanical properties of penta-graphene nanotubes. J. Phys. Chem. C.

[17] Wang Z.Y., Cao X.R., Qiao C., Zhang R.J., Zheng Y.X., Chen L.Y., Wang S.Y., Wang C.Z., Ho K.M., Fan Y.J. (2017). Novel penta-graphene nanotubes: Strain-induced structural and semiconductor-metal transitions. Nanoscale.

[18] Stauber T., Beltrán J.I., Schliemann J. (2016). Tight-binding approach to penta-graphene. Sci. Rep..

[19] Einollahzadeh H., Fazeli S.M., Dariani R.S. (2016). Studying the electronic and phononic structure of penta-graphane. Sci. Technol. Adv. Mater..

[20] Wang H., Maiyalagan T., Wang X. (2012). Review on recent progress in nitrogen-doped graphene: Synthesis, characterization, and its potential applications. ACS Catal..

[21] Agnoli S., Favaro M. (2016). Doping graphene with boron: A review of synthesis methods, physicochemical characterization, and emerging applications. J. Mater. Chem. A.

[22] Rani P., Jindal V.K. (2013). Designing band gap of graphene by B and N dopant atoms. RSC Adv..

[23] Berdiyorov G.R., Dixit G., Madjet M.E. (2016). Band gap engineering in penta-graphene by substitutional doping: First-principles calculations. J. Phys. Condens. Matter.

[24] Hohenberg P., Kohn W. (1964). Inhomogeneous electron gas. Phys. Rev..

[25] Kohn W., Sham L.J. (1965). Self-consistent equations including exchange and correlation effects. Phys. Rev..

[26] Sathishkumar N., Wu S.Y., Chen H.T. (2019). Boron- and nitrogen-doped penta-graphene as a promising material for hydrogen storage: A computational study. Int. J. Energy Res..

[27] Segall M.D., Lindan P.J.D., Probert M.J., Pickard C.J., Hasnip P.J., Clark S.J., Payne M.C. (2002). First-principles simulation: Ideas, illustrations and the CASTEP code. J. Phys. Condens. Matter..

[28] Clark S.J., Segall M.D., Pickard C.J., Hasnip P.J., Probert M.I.J., Refson K., Payne M.C. (2005). First principles methods using CASTEP. Z. Krist..

[29] Milman V., Winkler B., White J.A., Packard C.J., Payne M.C., Akhmatskaya E.V., Nobes R.H. (2000). Electronic structure, properties, and phase stability of inorganic crystals: A pseudopotential plane-wave study. Int. J. Quantum. Chem..

[30] Payne M.C., Teter M.P., Allen D.C., Arias T.A., Joannopoulos J.D. (1992). Iterative minimization techniques for ab initio total-energy calculations: Molecular dynamics and conjugate gradients. Rev. Mod. Phys..

[31] Perdew J.P., Burke K., Ernzerhof M. (1996). Generalized gradient approximation made simple. Phys. Rev. Lett..

[32] Monkhorst H.J., Pack J.D. (1976). Special points for Brillouin-zone integrations. Phys. Rev. B.

[33] Pickard C.J., Mauri F. (2001). All-electron magnetic response with pseudopotentials: NMR chemical shifts. Phys. Rev. B.

[34] Rajbanshi B., Sarkar S., Mandal B., Sarkar P. (2016). Energetic and electronic structure of penta-graphene nanoribbons. Carbon.

