# Supplementary material for: Ab-Initio Study of the Electronic and Magnetic Properties of Boron- and Nitrogen-Doped Penta-Graphene
Source: Nanomaterials (Basel). 2020 Apr 24;10(4):816. doi: 10.3390/nano10040816 (PMC7221657; doi:10.3390/nano10040816)
Supplement: Supplementary file 1 [file nanomaterials-10-00816-s001.pdf]

Supplementary Materials

Ab-Initio Study of the Electronic and Magnetic Properties of Boron- and Nitrogen-Doped Penta-Graphene

Chao Zhang [1,2,3,*], Yu Cao [1,2], Xing Dai [4], Xian-Yong Ding [1,2], Leilei Chen [1,2], Bing-Sheng Li [3] and Dong-Qi Wang [5,*]

[1] State Key Laboratory of Mining Response and Disaster Prevention and Control in Deep Coal Mines, Anhui University of Science and Technology, Huainan 232001, China; 2018200579@aust.edu.cn (Y.C.); dxy_vasp@163.com (X.Y.D.); 18255707001@163.com (L.L.C.)

[2] School of Materials Science and Engineering, Anhui University of Science and Technology, Huainan 232001, China

[3] State Key Laboratory for Environment-friendly Energy Materials, Southwest University of Science and Technology, Mianyang, Sichuan 621010, China; libingshengmvp@163.com (B.S.L.)

[4] Institute of Quantitative Biology and Medicine, SRMP and RAD-X, Soochow University, Suzhou 215123, China; daixing@suda.edu.cn (X.D.)

[5] Multidisciplinary Initiative Center, Institute of High Energy Physics, Chinese Academy of Sciences, Beijing 100049, China

* Correspondence: chaozhang@mail.bnu.edu.cn (C.Z.); dwang@ihep.ac.cn (D.Q.W.); Tel.: +86-554-6690-442 (C.Z.); Tel. +86-10-8823-6606 (D.Q.W)

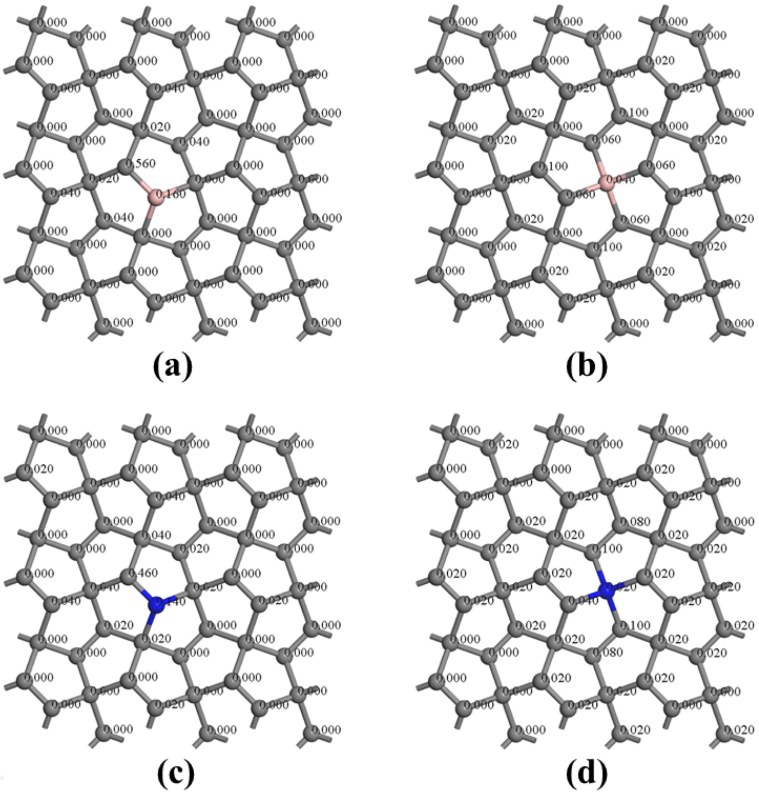

Figure S1. Atomic net magnetic moments (μ_B) for the (**a**) PG–B (sp^2), (**b**) PG–B (sp^3), (**c**) PG–N (sp^2) and (**d**) PG–N (sp^3) systems. Gray, pink and blue balls represent the carbon, boron, and nitrogen atoms, respectively.

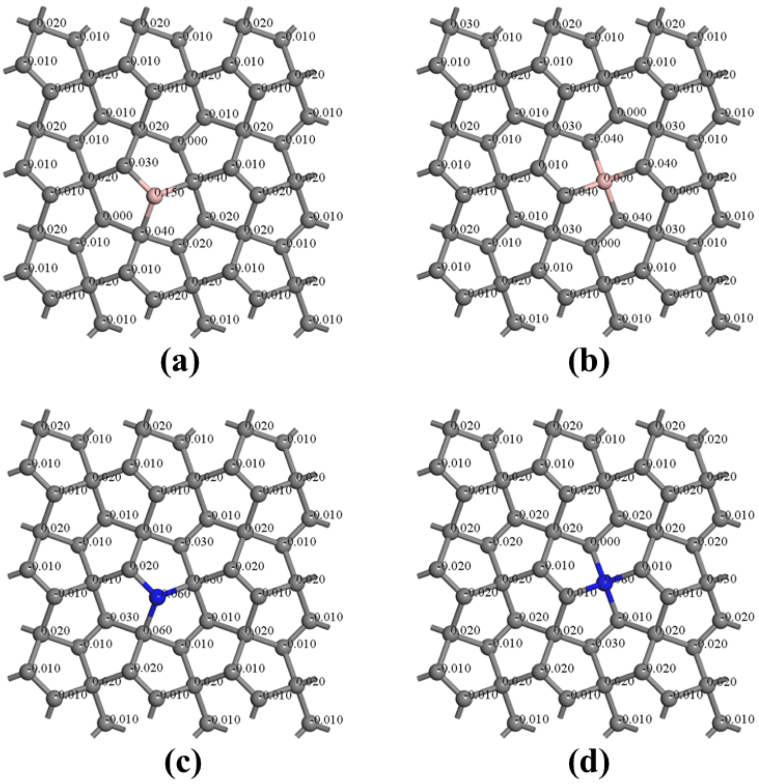

Figure S2. Atomic hirshfeld charge (|e|) for the (**a**) PG–B (sp^2), (**b**) PG–B (sp^3), (**c**) PG–N (sp^2) and (**d**) PG–N (sp^3) systems. Gray, pink and blue balls represent the carbon, boron, and nitrogen atoms, respectively.

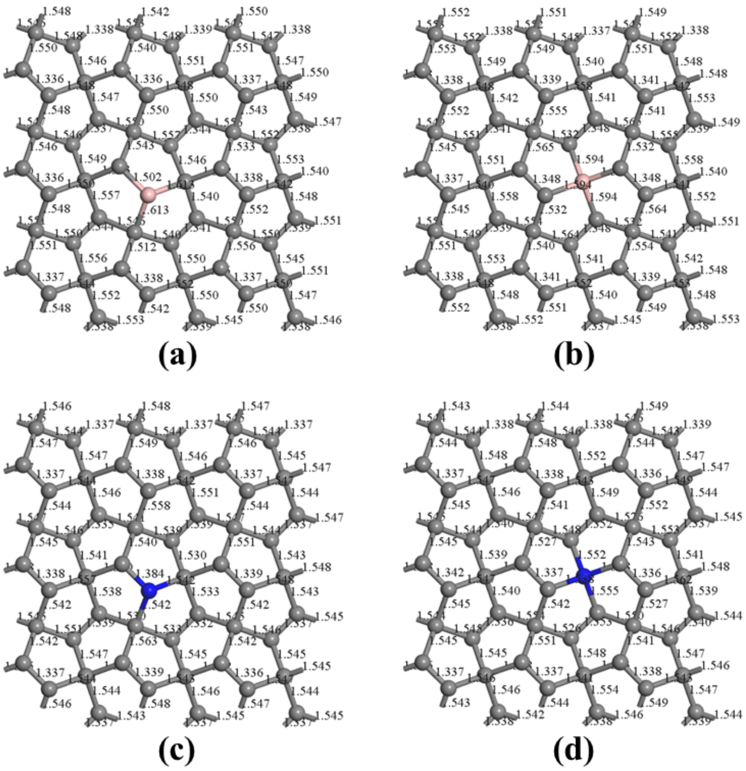

Figure S3. Bond lengths (Å) for the (**a**) PG–B (sp^2), (**b**) PG–B (sp^3), (**c**) PG–N (sp^2) and (**d**) PG–N (sp^3) systems. Gray, pink and blue balls represent the carbon, boron, and nitrogen atoms, respectively.
